# The Effect of Co-Enzyme Q10 on Acute Liver Damage in Rats, a Biochemical and Pathological Study

**DOI:** 10.5812/hepatmon.13685

**Published:** 2013-08-27

**Authors:** Soheil Ashkani Esfahani, Elmira Esmaeilzadeh, Fereshte Bagheri, Yasaman Emami, Mojtaba Farjam

**Affiliations:** 1 Student Research Committee, Shiraz University of Medical Sciences, Shiraz, IR Iran; 2 Department of Pharmacology, Fasa University of Medical Sciences, Fasa, IR Iran

**Keywords:** Liver, Rat, Pathology

*Dear Editor,*

Liver plays the role of detoxifier and excreter of destructive agents against body intoxication. Liver injury occurs following by histopathological changes including degeneration, necrosis, and atrophy of liver parenchymal cells with interstitial connective tissue as well as increasing in liver enzymes, such as aspartate aminotransferase/alanine aminotransferase (AST/ALT), alkaline phosphatase (ALP), and plasma total bilirubin level (TBili) (1). Acute Hepatic Encephalopathy (AHE) is described as a neuropsychiatric syndrome which can be pursued by urea cycle defect as well as acute (ALF) and chronic liver failure (CLF)(2). Clinical neuropsychiatric symptoms, such as attention impairment, memory loss, response inhibition, confusion, lethargy, and coma, can be found in these patients (3). Ammonium toxins releasing in blood as a result of liver dysfunction was reported to be a cause of AHE (4).

Coenzyme Q10 (CoQ10) is the key cofactor in electron transport chain. It was achieved that CoQ10 by its anti-oxidant, anti-inflammatory, and anti-apoptotic effects can have a therapeutic role in acetaminophen hepatotoxicity as well as metabolic-stress-induced liver damage (5, 6). Moreover, it was detected that CoQ10 could increase brain mitochondrial concentrations causing neuroprotective effects (7). In a study on an animal model of AHE, TAA induced liver damage, caused a reduction in plasma ammonia level, and slowed the progression into AHE condition in laboratory rats (8). The present study aimed to determine the hepatoprotective effects of CoQ10 against thioacetamide.

In this study, 36 Sprague Dawley rats (200-250gr) were divided into 3 groups (n=12): control group 1 (C1) which orally received 1cc normal saline daily starting from day 1, control group 2 (C2) which orally received 1cc normal saline daily starting from day 1, and experimental group (E) which received 50 mg/kg of CoQ10 PO daily from day 1 (the dosage was assigned according to a pilot study). On day 3, TAA (350 mg/kg) was intraperitoneally injected to all the animals, except of group C1. The last day of the study (day 10) was set as the day in which half or more than half of the rats in the C2 group reached grade IV of encephalopathy based on the neurobehavioral test scores (8). In order to evaluate the liver function, blood samples were obtained at the end and examined for the level of AST, ALT, ALP, and the level of plasma ammonia (NH4) by using clinical test kits (Randox, Randox Laboratories Ltd., UK). The degree of portal inflammation and hepatocellular necrosis were assessed qualitatively by an expert blinded pathologist and the changes were graded as following: absent, minimal, mild, and severe. Besides, neurobehavioral scoring was performed according to the method reported by Farjam et al. (8). Mann-Whitney U test was used for statistical analysis and all of the tests were done via SPSS® statistical software (17.0, IBM®, USA). In addition, P value ≤ 0.05 was considered as statistically significant.

The concentrations of liver enzymes (ALT, AST, ALP, and TBili) and NH4 are shown in Table 1. The study results indicated no significant difference between the E group and C1 group concerning the liver enzymes (ALT, AST, and ALP) and the ammonia level. However, a significant difference was observed between the E group and the C2 group in this regard (P<0.05). Histopathological examinations of the livers also described lower grades of portal inflammation (mild to moderate) and hepatocellular necrosis (mild to moderate) in the E group compared to the C2 group which both were in severe grades predominantly. Moreover, although the plasma level of NH4 in the E group was significantly higher than that of the C1 group, it was lower compared to the C2 group. Furthermore, the clinical grading of encephalopathy in the E group was lower in comparison with the C2 group. 

**Table 1.** The effect of CoQ10 on TBili (total bilirubin), ALT (alanine aminotransferase), AST (aspartate aminotransferase), ALP (Alkaline phosphatase), NH4 (plasma ammonia), and neurobehavioral grading in the animal model of Acute Liver Failure induced by TAA. Data is presented as Mean ± SD for control group 1 (C1), control group 2 (C2), and experimental group 1 (E1)

Groups	TBili (mg/dL)	AST (IU/L)	ALT (IU/L)	ALP (IU/L)	NH4 (µg/dL)	Neurobehavioral Grade
**C1**	1.2 ± 0.5	109.8 ± 53.2	122.7 ± 58.9	51.2 ± 30.7	198.9 ± 59.1	0
**C2**	0.9 ± 0.8	1262.7 ± 83.1^a^	778.3 ± 136.8^a^	898.5 ± 256.5^a^	1222.8 ± 83.6^a^	3.4 ± 0.55^a^
**E1**	0.9 ± 0.1	119.1 ± 25.8^b^	259.8 ± 111.5^b^	140.1 ± 76.2^b^	658.1 ± 272.6^a,b^	2.4 ± 0.55^a,b^

^a^ P ≤ 0.05 versus C1 group

^b^ P ≤ 0.05 versus C2 group

Previous studies showed the therapeutic effects of CoQ10 on the metabolic stress by inhibition of apoptosis in hepatocytes (6). Moreover, it was revealed that CoQ10 through its antioxidant, anti-inflammatory, and antiapoptotic effects could have a role in improvement of acetaminophen induced toxicity. These effects were assumed to be able to attenuate the cyclooxygenase activity (5). In this study, administration of CoQ10 in TAA-induced liver damage in rat models showed its beneficial effects as a hepatoprotective agent. CoQ10 ingestion also attenuated the neurobehavioral alterations caused by liver dysfunction. Finally, regarding the results of the present study and previous reports on CoQ10, this agent can be further evaluated for its positive impacts on the patients suffering from liver diseases. It may possibly be used as a supplement in order to attenuate the toxin induced complications.
